# Stillbirths at a hospital in Nablus, 2010: a cohort study

**DOI:** 10.3402/gha.v7.25222

**Published:** 2014-09-05

**Authors:** Tam Giao Cung, Anne Sofie Paus, Ammar Aghbar, Torvid Kiserud, Sven Gudmund Hinderaker

**Affiliations:** 1 Centre for International Health, University of Bergen, Bergen, Norway; 2 Rafidia Surgical Hospital, Nablus, Palestine; 3 Department of Obstetrics and Gynecology, Haukeland University Hospital, Bergen, Norway; 4 Department of Clinical Science, University of Bergen, Bergen, Norway

**Keywords:** stillbirth, Palestine, West Bank, Nablus, maternal haemoglobin, birth weight, macrosomia, prematurity

## Abstract

**Background:**

Stillbirths are insufficiently reported in many countries. In Nablus, reporting has recently started; little is published in Palestine on the stillbirth rate and its risk factors.

**Objective:**

To study the rate of stillbirths at Rafidia Hospital in 2010 and some of its risk factors.

**Design:**

A retrospective cohort design.

**Methods:**

Data were collected from the delivery registry for all births and we analysed those with a gestation of 28 weeks or more at Rafidia Hospital. Stillbirth rates were estimated for available determinants.

**Results:**

In 2010, a total of 5,644 women gave birth to 5,782 babies, of whom 41 were stillbirths, that is, a stillbirth rate of 7.1/1,000 births (95% confidence interval 5.2–9.5). Premature babies had a higher risk of being a stillbirth. For small babies, the lower the birth weight the higher was the probability of being a stillbirth, and for babies weighing 4,500 g or more there was a higher risk of being stillborn. The risk of stillbirth was also higher among babies from mothers with high haemoglobin concentration, but low maternal haemoglobin was not associated with stillbirths.

**Conclusions:**

The stillbirth rates at Rafidia hospital assessed in this study compares favourably with the reported national numbers, indicating a good reliability of the on-going registration. The rates were highest among premature births. Stillbirth was linked to low birth weight, foetal macrosomia, and maternal haemoconcentration. We believe the findings identify areas to address when designing antenatal care with the aim of improving perinatal mortality in the country.

It is estimated that there were 2.6 million stillbirths globally in 2009. Rates range from 3.1/1,000 births in high-income countries to 28.3 in sub-Saharan Africa (1). Intrapartum stillbirths represent 10–60%, with the highest proportion where stillbirth is more common (2). A stillbirth is frequently defined as the birth of a dead foetus that has reached 22 weeks of gestation or more, or has a birth weight of 500 g or more, but for international comparisons 28 weeks is more common as the lower limit of gestational age. Stillbirth statistics reflect both the quality of antenatal care and obstetric management during labour and childbirth, as well as factors in the environment, such as access to and quality of general healthcare, quality of life, and health-seeking behaviour (3, 4).

Many stillbirths are preventable through simple interventions, and a systematic registration of stillbirths is the first step towards improving health system planning (5). Causes of stillbirths can be foetal (e.g. congenital malformations, infections); placental (e.g. insufficiency, abruption); or maternal (e.g. infections, diabetes mellitus); but often the cause cannot be identified (2). Although perinatal mortality is of great interest to healthcare providers in middle- and low-income countries, many countries lack data on stillbirths (6). There is a need for research on stillbirths and its associated risk factors as a basis for developing interventions (2).

Nablus Governorate has started routine reporting of stillbirths, and in 2010, it reported the stillbirth rate to be 9.5/1,000 births, the highest in Palestine (7). Otherwise, little research has been done on stillbirths and its determinants in this region. Our aim was to study the stillbirths at Rafidia Hospital in Nablus in 2010, and our specific objectives were to 1) estimate the stillbirth rate and 2) assess the associations between stillbirths and available maternal and foetal determinants.

## Methods

### Design

The study had a retrospective cohort design.

### Setting

Nablus is situated on the West Bank and had around 340,000 inhabitants in 2010 (8). Nablus has been affected by the conflict between the Israeli Defence Forces and Palestinian groups. Unemployment and poverty is common, and restrictions to health access due to many checkpoints in the territory can be critical for both mother and foetus in obstetric emergencies. In 2010, Palestinian women were among the most educated in the Arab world with a female illiteracy rate of 7.4% above 15 years, and the total fertility rate was 3.8 on the West Bank (9). Antenatal care coverage was high, that is, 90% had ≥4 antenatal visits (10). Consanguinity has been and is still a common feature of Palestinian marriages (11). The crude birth rate on the West Bank in 2010 was 26/1,000 inhabitants (7).

In Nablus governorate, 99.5% of all live births took place in a hospital (7), often in crowded and understaffed government hospitals (12). There are six hospitals in Nablus, two of them governmental, Rafidia Surgical Hospital being the largest with a primary catchment area of an estimated 230,000 inhabitants. In 2010, this hospital registered more than half of the total number of reported births (9,565) in the whole Nablus governorate (7). Delivery in a government hospital such as Rafidia is free of charge for women in the catchment area.

### Data collection

The 2010 birth register from Rafidia Hospital was copied in July 2011. Our outcome measurement was ‘stillborn’. Stillbirths in our material were marked in the register as dead and with an Apgar score of zero, and we analysed foetuses of 28 weeks of gestation or more. The determinants were time of delivery, mother's age, parity, number of previous abortions, gestational age, haemoglobin concentration (Hb) at admission, mode of delivery, perineal tear, sex of the baby, and birthweight. A delivery was normal if vaginal and without use of forceps or vacuum extractor. A birth <37 weeks of gestation was defined as premature. Gestational age at Rafidia Hospital was usually determined by last menstrual period, or by ultrasound if in doubt.

### Analyses

All parameters were double entered into EpiData v.3.1 software (The EpiData Association, Odense, Denmark), checking and validating inconsistencies. Statistical analysis was done with EpiData Analysis v.2.2.2.178, except regression analysis which was done with IBM SPSS statistics version 19. Stillbirth rates were calculated for gestational age≥28 weeks and given per 1,000 births with 95% confidence limits, using OpenEpi calculator (13). Maternal haemoglobin level was categorised into five groups: Hb<70 g/L, Hb 70–89 g/L, Hb 90–109 g/L, Hb 110–144 g/L, and Hb≥145 g/L. Birth weight was categorised into four groups: <1,500 g; 1,500–2,499 g; 2,500–4,499 g; and ≥4,500 g. The association between stillbirth and risk factors was analysed by logistic regression, unadjusted and adjusted by forward stepwise (conditional) method.

### Ethical considerations

The project was approved by the Regional Committee for Medical and Health Research Ethics in Norway (2012/734/REK Vest) and local research permit was granted by the hospital director at Rafidia Surgical Hospital, as we found no local ethics committee in the area.

## Results

In 2010, 5,644 women gave birth at gestational age ≥28 weeks at Rafidia Hospital (Table 1); this resulted in 5,782 babies: 5,514 (95.4%) singletons, 244 (4.2%) twins, and 24 triplets (0.4%). The mothers had a mean age of 27 years, ranging from 15 to 48 years, and around a quarter of them gave birth for the first time. Anaemia (Hb <110 g/L) was observed in 2,142 mothers (37.0%) and high Hb (i.e. ≥145 g/L) in 53 (0.9%) women. In the same year, 2010, another 26 births had gestational age 22–27 weeks and 5 were stillbirths.

**Table 1:** Characteristics of 5,644 women delivering at a tertiary care hospital in Nablus, 2010

Total		Mothers, N	Mothers with stillbirths, n (%)
		5,644	38 (100)
Age	<20 y	380	1 (0.3)
	20–29 y	3,333	19 (0.6)
	30–39 y	1,716	15 (0.9)
	>40 y	214	3 (1.4)
	Not recorded	1	0 (0.0)
Parity at discharge	Para 1	1,338	8 (0.6)
	Para 2–3	1,991	12 (0.6)
	Para 4–5	1,411	5 (0.4)
	Para 6 or more	904	13 (1.4)
Previous abortions	None	4,164	27 (0.6)
	1–2	1,268	9 (0.7)
	3 or more	210	2 (1.0)
	Not recorded	2	0 (0.0)
Hb^a^ at admission	Hb<70 g/L	3	0 (0.0)
	Hb=70–89 g/L	195	1 (0.5)
	Hb=90–109 g/L	1,891	13 (0.7)
	Hb=110–144 g/L	3,502	22 (0.6)
	Hb≥145 g/L	53	53 (3.8)
Time of delivery^b^	Q1–2010	1,353	9 (0.7)
	Q2–2010	1,423	12 (0.8)
	Q3–2010	1,479	8 (0.5)
	Q4–2010	1,389	9 (0.7)

a Hb=maternal haemoglobin concentration.

b Q=calendar quarter.

There were 41 stillbirths recorded during the study period, resulting in a stillbirth rate of 7.1/1,000 births (for gestational age≥28 weeks). A total of 4,180 (72.3%) babies were born after a normal delivery. Caesarean section (CS) was done for 1,512 of all the babies (26.2%), among them were 14 (34%) stillbirths where indications were obstetric such as repeated sections, failed induction, or other maternal reasons. Stillbirth rates and risk of stillbirth by determinants are shown in Table 2. In unadjusted logistic regression, the risk of a stillbirth was six times higher among women with high Hb, whereas women with anaemia did not have increased risk. A stillbirth was much more common among babies with low birth weight than normal-sized babies, and babies with a high birthweight had almost six times higher risk of stillbirth. There were more stillbirths among premature than among term babies. In multivariable regression, significant associations remained among babies of low and high birth weight, premature babies, and babies of mothers with high Hb. The analysis showed that gestational age and birth weight contributed to 10% and 13% of the variation, respectively, being two distinct determinants.

**Table 2:** Stillbirth rates by maternal and foetal determinants among 5,782 babies born at a tertiary hospital in Nablus, 2010

Total		Babies born	Stillborn babies	Stillbirth/1,000 births (95% CI)	Crude odds ratio^a^ (95%CI)	Adjusted^a^,^b^ odds ratio (95%CI)
		5,782	41	7.1 (5.2–9.5)		
Maternal age	<20 y	391	1	2.6 (0.1–12.6)	0.3 (0.04–2.1)	
	20–29 y	3,407	21	6.2 (3.9–9.2)	Reference	
	30–39 y	1,768	16	9.0 (5.4–14.4)	0.7 (0.4–1.3)	
	>40 y	215	3	14.0 (3.6–37.5)	0.4 (0.4–5.4)	
	Missing	1	0	0	–	
Parity at discharge	Para 1	1,367	8	5.9 (2.7–11.1)	0.8 (0.3–1.9)	
	Para 2–3	2,042	15	7.3 (4.3–11.8)	Reference	
	Para 4–5	1,443	5	3.5 (1.3–7.7)	0.5 (0.2–1.3)	
	Para 6 or more	930	13	14.0 (7.8–23.2)	1.9 (0.9–4.0)	
Previous abortions	None	4,269	29	6.8 (4.6–9.6)	Reference	
	1–2	1,299	10	7.7 (3.9–13.7)	1.1 (0.6–2.3)	
	3 or more	212	2	9.4 (1.6–30.8)	1.4 (0.3–5.9)	
	Not recorded	2	0	0	–	
Hb at admission	Hb <70 g/L	3	0	0	–	–
	Hb=70–89 g/L	203	1	4.9 (2.5–24.1)	0.8 (0.1–5.7)	0.6 (0.1–4.5)
	Hb=90–109 g/L	1,936	15	7.7 (4.5–12.5)	1.2 (0.6–2.3)	1.0 (0.5–1.9)
	Hb=110–144 g/L	3,587	23	6.4 (4.2–9.5)	Reference	Reference
	Hb≥145 g/L	53	2	37.7 (6.3–119.1)	**6.1 (1.4**–**26.4)**	**9.6 (2.1**–**44.2)**
Quarter of the year	Q1–2010	1,386	9	6.5 (3.1–11.9)	Reference	
	Q2–2010	1,447	12	8.3 (4.5–14.1)	1.3 (0.5–3.0)	
	Q3–2010	1,515	9	5.9 (2.9–10.9)	0.9 (0.4–2.3)	
	Q4–2010	1,434	11	7.7 (4.0–13.3)	1.2 (0.5–2.9)	
Mode of delivery	Normal	4,180	27	6.5 (4.3–9.3)	Reference	
	Caesarean section	1,512	14	9.3 (5.3–15.1)	1.4 (0.8–2.7)	
	Vacuum extraction	41	0	0	–	
	Forceps extraction	9	0	0	–	
	Missing	40	0	0	–	
Sex of baby	Male	2,976	20	6.7 (4.2–10.2)	Reference	
	Female	2,805	21	7.5 (4.8–11.2)	1.1 (0.6–2.1)	
	Missing value	1	0	0	–	
Gestational age	28–36 w	373	20	53.6 (34.0–80.2)	**15.0 (8.0**–**28.1)**	**3.9 (1.4**–**10.4)**
	37–41 w	5,308	20	3.8 (2.4–5.7)	Reference	Reference
	42 w or more	73	1	13.7 (0.7–65.7)	3.7 (0.5–27.7)	4.0 (0.5–31.1)
	Missing value	28	0	0	–	
Birth weight	<1,500 g	69	11	159.4 (86.8–260)	**57.8 (25.9**–**128.7)**	**21.2 (6.6**–**67.9)**
	1,500–2,499 g	413	11	26.6 (14.1–45.8)	**8.3 (3.9**–**17.9)**	**4.1 (1.5**–**11.3)**
	2,500–4,499 g	4,580	16	3.5 (2.1–5.6)	Reference	Reference
	≥4,500 g	720	3	4.2 (1.1–11.3)	**5.9 (1.3**–**25.7)**	**6.3 (1.4**–**27.8)**
Multiple pregnancy	Singletons	5,514	38	6.9 (5.0–9.4)	Reference	
	Twins	244	3	12.3 (3.1–33.1)	1.8 (0.6–5.8)	
	Triplets	24	0	0	–	

a Bold type indicates odds ratios (OR) significantly differing from unity.

b Forward stepwise conditional inclusion in logistic regression.

## Discussion

In this study from a large hospital in Nablus, at which more than half of all deliveries in the area take place, we found that 7.1‰ (95% CI 5.2–9.5) were stillborn, which is not statistically different from the official numbers of the governorate of 9.5‰ (7), but different from the 3.6‰ reported from the West Bank, the latter possibly reflecting incomplete reporting. With a very high proportion of the population's reported deliveries taking place in the hospitals (7), we believe that our results reflect fairly well the rate of stillbirths in this population. The estimated stillbirth rates in Jordan and Egypt (13 and 10‰, respectively) in 2006 were comparable, whereas neighbouring Israel had a lower rate, 5‰ (6). We also believe that the present study by independently replicating the results of the governmental registry confirms their reliability.

A strength of our study was that we used data from a real-life situation registered at a large maternity unit covering a sizable population. A register is also a potential weakness, as some errors will occur during the registration process, and some variables can be missing. Transcribing and digitalizing the data can also introduce errors, but this was controlled for by repeat entry. Incomplete recording cannot be excluded, but the fact that the present results corresponded well with official reports on stillbirths suggests that the number is small. Some associations appear statistically significant but rely on very small numbers of stillbirths (e.g. high maternal Hb); here interpretation must be done with caution. Furthermore, even though almost all deliveries in Nablus take place in health facilities making their registers fairly representative for the population, those few who do not may be a selected group of higher risk for adverse outcomes, causing a possible underestimation of the stillbirth rate in the present study. Similarly, an unknown difference in preference of admission to various institutions may affect risk distribution and estimates. Although we believe the present estimate is a fair representation for the population, we acknowledge that the study is not strictly population based and therefore has to be regarded as an approximation.

In our study, the pregnant women with high Hb had six times higher risk of having a stillborn baby, but this condition was observed in less than 1% and is therefore not a prominent public health problem. On the other hand, low Hb was a common condition in this population (37%) but not a significant risk factor for stillbirth as observed in other studies (14, 15). It may raise the question whether the cut off of Hb<110 g/L for anaemia in pregnancy endorsed by WHO is set too high for the present study population. Hb falls during pregnancy due to physiological dilution reaching a minimum in early third trimester, leading to misclassifications in the present study where Hb was taken at admission for birth.

High Hb concentration is also known to be associated with pre-eclampsia (16), that is, placental insufficiency and impaired foetal growth. However, the birth register had no information about blood pressure and previous pre-eclampsia to further explore this link. Adding such information would improve the utility of the register for research on common causes of maternal morbidity, foetal growth restriction, prematurity, and stillbirth.

Small and large babies were associated with increased risk of stillbirth in line with other studies (17, 18). Stillbirth occurred more commonly among prematurely born, and their birth weight was lower than their peers. Although this finding is in line with previous reports showing that growth-restricted foetuses have an increased risk of stillbirth (19, 20), the uncertainty of birth weight has to be kept in mind since the weight of the foetus falls after death, and this reduction increases with time. In our cases, we had no information time of foetal death. Identifying pregnancies at risk of developing foetal growth restriction and pre-eclampsia (e.g. having previously had pre-eclampsia or growth restricted baby) could lead to a more focused follow up, including ultrasound monitoring of growth and thus timely intervention to avoid intrauterine foetal death. The largest babies also had an increased risk of stillbirth, although almost all of them were full term and also had an increased risk of stillbirth. Hyperglycaemia, particularly when combined with macrosomia (21), has an increased risk of intrauterine death, but macrosomia is also associated with obstructed labour with ensuing complications, including fresh stillbirth. Adding information on maternal height and weight (for BMI) could help assess the health and nutritional status of the pregnant population and its risks for developing diabetes, hypertension, and other pregnancy-related complications such as stillbirth (22).

More information on when the baby ceased quickening and a more detailed assessment of the neonate concerning maceration and other signs would help identify intrapartum deaths that are more likely to be prevented by timely interventions.

We also acknowledge the value of recording the 5 stillbirths among 26 births (19%) between 22 and 28 weeks of gestation. They constitute an important supplement giving a more complete view of perinatal care, showing that even this early stage of pregnancy needs further attention to identify which of these deaths are due to preventable conditions.

## Conclusions

In this study from the Palestinian territory, we found a rate of stillbirth of 7.1‰, which is in line with the reported figures from these areas. Prematurity, small foetuses, foetal macrosomia, and maternal haemoconcentration were significant risk factors and could be considered when designing the strategy of antenatal care with the aim of reducing stillbirths (and perinatal mortality) in the population. We believe that including information on maternal height and weight, blood pressure, and albuminuria in addition to available information on hyperglycaemia would enhance the registry's value as a tool for designing antenatal care and intrapartum monitoring. There is also a need to understand more precisely how many stillborns are due to intrapartum death.
